# Efficacy and safety of moxibustion in the treatment of infertility with polycystic ovary syndrome: A protocol of systematic review and meta-analysis

**DOI:** 10.1097/MD.0000000000024529

**Published:** 2021-03-19

**Authors:** Wenguo Ye, Gen Deng, Lin Yin, Jing Ye

**Affiliations:** a Affiliated Hospital of Jiangxi University of Traditional Chinese Medicine; b Jiangxi University of Traditional Chinese Medicine, Nanchang, China.

**Keywords:** infertility, moxibustion, polycystic ovary syndrome (PCOS), protocol, systematic review and meta-analysis

## Abstract

**Background:**

Polycystic ovary syndrome (PCOS) is one of the common diseases of reproductive endocrine metabolism in gynecology, and it is also a common and difficult disease affecting female reproductive endocrine health. PCOS characterized by insulin resistance and hyperandrogenemia, the clinical manifestations are polychaemia, acne, obesity, infertility, menstrual disorders and so on. Clinical treatment of patients with PCOS ovulatory dysfunction infertility is mainly treated with ovulation-promoting drugs, insulin sensitizer, hyperandrogenemia drugs and other drugs Healing. It is found that the sensitivity of patients to ovulation promotion is poor, and it is often necessary to increase the dosage of drugs to increase ovulation rate, thus increasing the risk of ovarian hyperstimulation syndrome, and the recurrence rate is higher after withdrawal. Moxibustion therapy has shown strong advantages in the treatment of PCOS, and the curative effect is accurate. Therefore, this paper will carry out a systematic evaluation and meta-analysis of the efficacy and safety of moxibustion therapy in the treatment of PCOS.

**Methods:**

We will search 8 electronic databases, including PubMed, Embase, Web of Science, Cochrane Library, the China National Knowledge Infrastructure (CNKI), Chinese Science and Technology Periodical Database (VIP), Wanfang Database (WF), and Chinese Biomedical Literature Database (CBM). We will search above electronic databases from the beginning to January 2021, without any language restriction. Ovulation rate and pregnancy rate will be accepted as the primary outcomes. The changes of Sex hormone levels, including Luteinizing hormone, follicle-stimulating hormone, serum estradiol, total testosterone will be used as secondary outcomes. RevMan 5.3 software will be used for statistical analysis. The result about the curative effect and safety of moxibustion therapy for PCOS will be presented as risk ratio for dichotomous data and mean differences with a 95% confidence interval for continuous data.

**Results:**

Only when we finish this meta-analysis can we get the result.

**Conclusions:**

The results of this study will provide reliable evidence for the efficacy and safety of moxibustion therapy in the treatment of PCOS.

## 1 Introduction

Polycystic ovary syndrome (PCOS) is one of the common diseases of reproductive endocrine metabolism in gynecology, and it is also a common and difficult disease affecting female reproductive endocrine health.^[1]^ PCOS is characterized by insulin resistance and hyperandrogenemia. the clinical manifestations are polychaemia, acne, obesity, infertility, menstrual disorder and so on.^[2]^ Studies have shown that this disease affects 6% to 21% of women in the world,^[3]^ and has a significant impact on women's life. PCOS is one of the main causes of anovulatory infertility, which accounts for 25% to 30% of anovulatory infertility,^[4]^ the incidence is about 5.6% in China.^[5]^ Data show that treatment PCOS infertile IVF alone costs about 100 billion.^[6]^ PCOS assisted pregnancy has become a difficult point in the treatment of infertility.^[7]^ With the development of PCOS pathogenesis, ovulation promotion drug therapy and insulin sensitizer therapy have become the focus of PCOS pregnancy therapy. At present, the clinical treatment PCOS ovulation dysfunction infertility patients are mainly treated with ovulation promotion drugs, insulin sensitizer, hyperandrogenemia drugs and other drugs. Some studies have found that patients are less sensitive to proovulation drugs, It is often necessary to increase the dosage of drugs to increase ovulation rate, thus increasing the risk of ovarian hyperstimulation syndrome, and the recurrence rate is higher after withdrawal.^[8]^ Due to the long time, high price, many adverse reactions, protruding side effects, low pregnancy rate, poor curative effect and drug resistance, it has become a difficult problem to be solved in clinic.^[9]^ In recent years, literatures on moxibustion therapy for urticaria have been increasing year by year. However, there is still a lack of systematic evaluation on the efficacy and safety of moxibustion therapy for PCOS in clinical practice. Therefore, the effectiveness and safety of moxibustion therapy in the treatment of PCOS will be systematically evaluated and meta-analyzed in this paper.

## 2 Methods

### 2.1 Study registration

This protocol was registered with the International Platform of Registered Systematic Review and Meta-Analysis Protocols (INPLASY) on January 01, 2021 and was last updated on January 01, 2021 (registration number INPLASY202110004).

### 2.2 Inclusion criteria for study selection

#### 2.2.1 Types of studies

Clinical randomized controlled trials (RCTs) containing moxibustion therapy for infertility with PCOS were included, with no limitation of language and publication status.

#### 2.2.2 Types of participants

There are clear and recognized diagnostic criteria and efficacy criteria, and all patients are diagnosed as PCOS, regardless of gender, age and origin of the case.

#### 2.2.3 Types of interventions

##### 2.2.3.1 Experimental interventions

Moxibustion therapy includes all combination therapies using any type of moxibustion therapy or based on moxibustion therapy in combination with other therapies.

##### 2.2.3.2 Control interventions

The control group will receive one of the following treatment methods: conventional pharmacological therapy, no treatment, and placebo.

#### 2.2.4 Types of outcome measures

##### 2.2.4.1 Primary outcome

Moxibustion therapy, or mixed therapies based on moxibustion therapy will also be include.

##### 2.2.4.2 Secondary outcomes

The changes of sex hormone levels, including Luteinizing hormone, follicle-stimulating hormone, serum estradiol, total testosterone will be used as secondary outcomes.

### 2.3 Exclusion criteria

Non-randomized controlled trials; no exact diagnostic scale or therapeutic scale; no moxibustion therapy as the main treatment in the experimental group, and moxibustion therapy was found in the control group. Repeated literature; theory and review literature; animal experiments; nursing research.

### 2.4 The retrieval methods and strategies of this study

#### 2.4.1 Electronic database retrieval

We will search 8 electronic databases, including PubMed, Embase, Web of Science, Cochrane Library, the China National Knowledge Infrastructure (CNKI), Chinese Science and Technology Periodical Database (VIP), Wanfang Database (WF), and Chinese Biomedical Literature Database (CBM). We will search above electronic databases from the beginning to January 2021, without any language restriction. And will searching the relevant literature by combining subject words with free words, search terms consist of disease (“polycystic ovary syndrome” or “PCOS” or “Ovary Syndrome, Polycystic” OR “Stein Leventhal Syndrome” OR “Sclerocystic Ovarian Degeneration”) and intervention (“moxibustion” or “moxa” or “moxabustion” or “cauterize” or “mugwort”) and research types (“randomized controlled trial” or “controlled clinical trial” or “random trials” or “RCT”or “RCTS”). The PubMed search strategy is shown in Table 1.

**Table 1:** PubMed retrieval strategies.

ID	Query
#1	“polycystic ovary syndrome”[Mesh]
#2	(((((Ovary Syndrome, Polycystic[Ti/Ab]) OR (Stein Leventhal Syndrome[Ti/Ab])) OR (Sclerocystic Ovarian Degeneration[Ti/Ab])) OR (Syndrome, Polycystic Ovary[Ti/Ab])) OR (Ovarian Degeneration, Sclerocystic[Ti/Ab])) OR (PCOS[Ti/Ab])
#3	#1 OR #2
#4	“moxibustion”[Mesh]
#5	((((Moxabustion[Ti/Ab]) OR (moxa[Ti/Ab])) OR (moxabustion[Ti/Ab])) OR (cauterize[Ti/Ab])) OR (mugwort[Ti/Ab])
#6	#4 OR #5
#7	(((randomized controlled trial[Ti/Ab]) OR (random trials[Ti/Ab])) OR (controlled clinical trial[Ti/Ab])) OR (RCT[Ti/Ab])
#8	#3 AND #6 AND #7

Ab = abstract, Mesh = medical subject headings, Ti = title.

#### 2.4.2 Searching other resources

We will combine manual retrieval of literature resource database to search relevant conference papers that meet the inclusion criteria. In addition, the grey literature, as well as ongoing and recently completed studies, will be searched on Clinicaltrials.gov.

### 2.5 Data extraction and management

#### 2.5.1 Literature inclusion and data extraction

**Figure 1:**
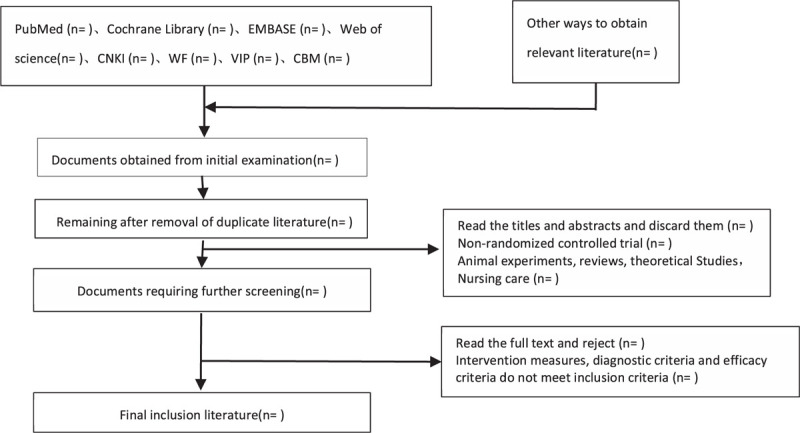
Literature screening flow chart.

The 2 researchers independently read the title and abstract of the literature we obtained, read the full text of the trials that might meet the inclusion criteria to determine whether the inclusion criteria were truly met, and discussed the conflicting literatures or let the third researcher decide whether to include them. Two researchers independently extracted data from the included studies, including study design, intervention measures and methods, measurement indicators, results, methodological contents such as hidden grouping and blind method, etc., and a third evaluator checked the consistency of the data. If the required information is incomplete, we will contact the original author for the required data. The inclusion process of this study will be carried out as shown in Figure 1.

#### 2.5.2 Methodological quality evaluation

Two evaluators independently select the literature according to the inclusion and exclusion criteria and cross-check. In case of disagreement, a third evaluator will assist in the decision. The extracted data included the first author, year of publication, number of patients, age, gender, intervention measures, outcome indicators, etc. The Jadad scale to evaluate quality into literature, including: random sequence (right 2 points, 1 points not clear, inappropriate 0), distribution, hidden (right 2 points, 1 points not clear, inappropriate 0), blinded (right 2 points, 1 points not clear, inappropriate 0), lost to follow-up and exit (describe 1 points, not describe 0); 0 to 3 is classified as low quality and 4 to 7 as high quality.

### 2.6 Statistical analysis

#### 2.6.1 Quantitative data synthesis

Meta-analysis will be performed using Rev Man5.3 software. The odds ratio (OR) and its 95% confidence interval (CI) will be used as the counting data, while the weighted mean difference (WMD) and its 95% CI will be used as the measurement data.

#### 2.6.2 Assessment of heterogeneity

The heterogeneity test will be carried out first among all studies, *I*^2^ test will be used. When *P* > .1 and *I*^2^ < 50%, the fixed effect model will be used; otherwise, the random effect model will be used. When the clinical heterogeneity between the 2 studies is large, only descriptive analysis will be performed.

#### 2.6.3 Publication bias

When the number of qualified RCTS is sufficient, we will use the inverted funnel Egger to test the potential publication bias.

#### 2.6.4 Subgroup analysis

If heterogeneity exists in the meta-analysis, the source of heterogeneity should be sought, such as whether the degree of disease, treatment cycle, treatment time of each moxibustion, type of intervention, etc., is the source of heterogeneity. If so, a subgroup analysis should be conducted for these reasons to see whether heterogeneity still exists after analysis.

#### 2.6.5 Sensitivity analysis

Sensitivity analysis can not only assess the stability and reliability of the combined results, but also assess whether the combined results are significantly changed by the influence of a single study. If sufficient literature is included, we will adopt the method of excluding literature one by one, excluding each included study one by one before effect-size combination, changing the inclusion and exclusion criteria or excluding certain types of literature before effect-size combination.

## 3 Discussion

The symptoms of PCOS could be summarized as “infertility”, “Amenorrhea”, “lump” and “leakage” in Traditional Chinese medicine. The TCM etiology and pathogenesis of this disease is believed to be closely related to dysfunction of the kidney, liver and spleen, phlegm dampness and blood stasis.^[10]^ Modern Traditional Chinese medicine believes that the key link of the disease is the kidney – decyl – chongren – cellular reproductive axis dysfunction and dysfunction.^[11]^ Previous studies conducted statistical analysis of TCM syndromes of PCOS, and found that kidney deficiency and blood stasis, phlegm-dampness block, kidney deficiency and phlegm-dampness are the main syndromes, and the frequency of kidney deficiency syndrome is the highest, which is consistent with the pathogenesis characteristics of kidney deficiency.^[12]^ It can be seen that this disease is based on kidney deficiency, involving the liver and spleen, phlegm and dampness, blood stasis as the main pathological factors, and the 2 influence each other, and eventually lead to renal – tiangui – Chongren – dystonic axis imbalance and morbidity.^[13]^ The treatment of ovulation-promoting drugs after the traditional Chinese medicine protocol pretreatment can optimize the treatment effect, effectively improve the pregnancy rate, efficacy and safety.^[14]^ At present, there are various methods of acupuncture and moxibustion to treat PCOS, including simple acupuncture, electroacupuncture therapy, moxibustion therapy, acupoint embedding, auricular acupoint therapy, acupuncture and medicine usage, etc. Some studies believe that acupuncture and moxibustion can improve the patients’ ovarian microenvironment, sympathetic nervous system, endocrine system and glucose and lipid metabolism disorder, and can improve the patients’ psychological state, with significant curative effect and small adverse reactions.^[15]^ Therefore, it is necessary to systematically evaluate the treatment of PCOS by moxibustion therapy in this study, which can provide evidence-based medicine evidence for future clinical guidance of the treatment of PCOS by moxibustion therapy.

## Author contributions

**Data curation:** Jing Ye, Gen Deng.

**Formal analysis:** Jing Ye, Gen Deng.

**Investigation:** Gen Deng, Jing Ye.

**Methodology:** Gen Deng, Lin Yin.

**Project administration:** Jing Ye, Wenguo Ye.

**Software:** Gen Deng, Lin Yin.

**Supervision:** Wenguo Ye.

**Validation:** Wenguo Ye.

**Visualization:** Lin Yin.

**Writing – original draft:** Jing Ye, Wenguo Ye.

**Writing – review & editing:** Jing Ye, Wenguo Ye.
